# Assessment of Soft Actuators for Hand Exoskeletons: Pleated Textile Actuators and Fiber-Reinforced Silicone Actuators

**DOI:** 10.3389/fbioe.2022.924888

**Published:** 2022-07-12

**Authors:** Orion Ramos, Marcela Múnera, Mehran Moazen, Helge Wurdemann, Carlos A. Cifuentes

**Affiliations:** ^1^ Biomedical Engineering Department, Department of Biomedical and Electronic Engineering, Colombian School of Engineering Julio Garavito, Bogota, Colombia; ^2^ Department of Mechanical Engineering, University College London, London, United Kingdom; ^3^ School of Engineering, Science and Technology, Universidad del Rosario, Bogota, Colombia; ^4^ Bristol Robotics Laboratory, University of the West of England, Bristol, United Kingdom

**Keywords:** assessment, soft actuators, soft robotics, fiber-reinforced, textile actuator, hand exoskeleton

## Abstract

Soft robotic approaches have been trialed for rehabilitation or assistive hand exoskeletons using silicone or textile actuators because they have more tolerance for alignment with biological joints than rigid exoskeletons. Textile actuators have not been previously evaluated, and this study compares the mechanical properties of textile and silicone actuators used in hand exoskeletons. The physical dimensions, the air pressure required to achieve a full bending motion, and the forces generated at the tip of the actuator were measured and compared. The results showed that the construction method of the silicone actuators is slower than the textile actuators, but it generates better dimensional accuracy. However, the air pressure required for the actuators to generate a full bending motion is significantly lower for textile actuators, and the blocking force generated at that pressure is 35% higher in the textile actuators. There are significant differences across all variables compared, indicating that actuators constructed using pleated textile techniques have greater potential for the construction of an exoskeleton for hand rehabilitation or assistance.

## 1 Introduction

In the United States, 2.8 million stroke were recorded in 2018 (Center for Health Statistics, 2018; Abbasi et al., 2020), and only seven millions of people are stroke survivors (Foundation, 2021). Half of stroke survivors are left with a type of paralysis that affects their ability to perform basic activities of daily living (ADL). Medically, this paralysis is treated by rehabilitation therapies that involve progressive and repetitive movements that eventually restore mobility and strength to the affected limbs. These rehabilitation strategies include supervision by a health expert who traditionally performs the therapy and manages the repetitive movements during the rehabilitation session. However, patient adherence to this type of therapy is low due to its high financial cost and slow results (Takahashi et al., 2007). Over the years, robotic solutions have been developed to improve therapies and the living conditions of stroke survivors. Rehabilitation procedures have integrated devices such as exoskeletons that facilitate therapies by helping to perform repetitive movements and to quantify patients’ progress. Patients undergoing rehabilitation therapy using robotic technologies are shown to have better adherence to programs and significant improvements in motor function (Kutner et al., 2010).

Of the limbs affected with movement paralysis after stroke, the hand is typically one of the last limbs to be rehabilitated. Compared to the lower limbs, the hand requires less force to assist its movements, but it has a greater range of movement (Agarwal et al., 2016). When robotic devices are used, the design of exoskeletons for hand rehabilitation is significantly more complex due to this large capacity of the human hand in terms of dexterity and the number of degrees of freedom (Gabardi et al., 2018). Designers of hand exoskeletons must ensure that each joint of the human finger can be controlled independently, thus providing adequate mobility to perform daily tasks (Hasegawa et al., 2011).

The development of these devices evolved from rigid technologies where the exoskeletons were driven by mechanisms based on gears and motors located in the finger joints (Aiple and Schiele, 2013). This methodology involves ensuring that the dimensions of the links were exactly fitted to the finger to be rehabilitated to avoid exerting unnecessary force (Li et al., 2011). The correct position and design prevent damage or distortion of the fingers. The creation of soft technology has facilitated the design of these devices by eliminating mechanisms that require exact alignment of the limb joints with the degrees of freedom of the exoskeleton (Agarwal et al., 2016). These technologies also help to reduce the weight of the hand during rehabilitation by moving the power source for actuation to an external location, such as a backpack or control box (Bützer et al., 2021). Within this type of technology, different devices have been developed based on motor-driven tendons that can perform a rehabilitation session for extension-flexion and abduction-adduction of the thumb (Yap et al., 2017b). Pneumatic actuators are another type of technology used to create rehabilitation devices and hand assistance based on the soft actuator paradigm. This technology, like the tendon-drive, allows separate sources of actuation outside of the hand to reduce the weight of the device (Agarwal et al., 2016). In general, the design of hand rehabilitation and assistive devices aims to reduce the system’s weight without sacrificing the device’s functionality (Jiang et al., 2017).

The requirements for designing an assistive device for hand rehabilitation are currently divided into three categories (Polygerinos et al., 2015a). The first includes the practical considerations, such as the number of fingers to be assisted, and the weight and dimensions of the device. Second, the necessary kinematic requirements for the device to be considered functional in clinical rehabilitation or assistive environments are defined. This category defines the ranges of motion and forces that are necessary for each actuator of the exoskeleton. Finally, the control category specifies parameters such as the response speed of the actuators, the system’s bandwidth, and the sensing of the device. For each of the categories, values and requirements are defined based on engineering studies and clinical recommendations.

Regarding practical considerations, the total weight of the wearable device should not exceed 3 kg, and the weight supported in hand should be around 0.5 kg (Polygerinos et al., 2015a). The actuator dimensions should be in the range of the size of human fingers (Bundhoo and Park, 2005). For the kinematic requirements, each actuator must bend at least 250° in the bending angle to be considered able to assist in human finger flexion (Polygerinos et al., 2015a). The force to be exerted by the actuators is set higher than 7 N at the distal tip to allow any assistance in ADL. This value is calculated for objects that do not weigh more than 1.5 kg (Polygerinos et al., 2015a), but only 3 N of force generated by each actuator is sufficient to generate functional exercises for hand rehabilitation (Agarwal et al., 2016). For control aspects, the sampling rate must be at least 20 times faster than the actuator response rate (Polygerinos et al., 2015a). Different methods have been established to define the actuation time of actuators used in hand exoskeletons, and this time varies depending on the task and the type of device. For example, devices must execute the closing and opening movements in less than 20 s (Li et al., 2019), (Borboni et al., 2016). The generation of the grip in assistive devices requires the actuators to generate the grip in less than 4 s (Yap et al., 2017b), (Tran et al., 2020), and the closing time should track to that of a healthy hand, which is 1–3 s (Boser et al., 2020), (Mohammadi et al., 2018). Finally, the system must be powered by a small power supply and air source while staying within the total allowable weight (Masia et al., 2018).

Wearable robotics and soft robotics techniques have inspired different types of devices for hand rehabilitation. In particular, the types of actuators most commonly used for the construction of assistive and rehabilitative hand devices are fiber-reinforced (FR) silicone actuators and textile actuators. An exoskeleton for rehabilitation and task-specific training using only the FR-type actuator was presented in 2015 (Polygerinos et al., 2015a). This device is unusual in that each actuator controls different sections of the movement, allowing the actuator to generate assistance in the bending and twisting of the fingers. The functionality of this device was evaluated by the Kapandji test, and box and block test (Polygerinos et al., 2015a). In 2017, the fabrication method of the exoskeleton was modified to make the actuators more robust. These actuators performed correctly for 62.2 cycles in an inflation and deflation test at 100 kPa (Jiang et al., 2017). Each device’s actuator weighs 37 g, which was a factor identified for improvement in future versions (Heung et al., 2019).

Exoskeletons built with textile-type actuators usually assist in the bending and extending of the fingers by using layers of fabrics that have different properties. A hand exoskeleton with textile actuators composed of flexible materials, such as fabric with a thermoplastic polyurethane (TPU) coating, was constructed in 2017 and found to help in bending the fingers (Yap et al., 2017a). However, the force generated in extension was not enough for patients with high muscle spasticity (Yap HK. et al., 2017). The following year, an exoskeleton was built with textile actuators with pleats that facilitate the bending motion and reduce the input pressure, allowing the device to assist in opening and closing the hand (Cappello et al., 2018). This version of the device requires 25 psi of power to generate different grips (Zhou et al., 2019).

To determine which type of actuator is better in the construction of assistive or rehabilitative hand devices based on the design requirements, the performance of the actuators was evaluated and compared using a series of mechanical tests. The variables investigated included the pressure required to generate the maximum bending (Mosadegh et al., 2014) and the force that the actuators can generate (Keong and Hua, 2018). To evaluate the maximum bending, a test measures the air pressure to the maximum bending angle at the tip of the actuator. In 2013, a comparative actuator tip pressure vs. actuator tip angle test reported that it is possible to achieve mean bending at pressures in the range of 42–52 kPa, depending on the actuator length (Polygerinos et al., 2013). In the same year, a comparison of silicone actuators was carried out in which it was found that an FR actuator made of Elastosil M4601 material with a length of 16 cm achieves full bending at 243 kPa (Galloway et al., 2013). In 2015, a study was conducted to identify how construction parameters such as the actuator length, the internal air chamber’s internal radius, and the actuator wall thickness affect the pressure required to achieve full bending. It concluded that the smaller the actuator length and internal radius, the higher the required pressure. In addition, the chamber wall is directly proportional to the required pressure, so the thicker the actuator, the higher the pressure. In the same study, an actuator with a length of 16 cm, a wall thickness of 2 mm, and an inner radius of 8 mm was constructed, allowing full bending to be achieved at 200 kPa (approximately 30 psi) (Polygerinos et al., 2015b). Another study reported that 300 kPa was necessary to achieve half bending with an FR silicone actuator (Nordin et al., 2016). Finally, in 2018, silicone actuators achieved medium bending (close to 90° bending) at a pressure of 110 kPa. It was also shown that the actuator reduces this angle if attached to a finger simulating an exoskeleton (Ariyanto et al., 2018). As seen in these studies, variation in actuator dimensions, the material of construction, and the type of reinforcement change the actuator’s behavior concerning the required input pressure that generates the maximum deflection.

Finding the force generated by soft actuators is done in several ways. The most common technique measures the actuator tip force with a load cell (Abbasi et al., 2020). Two configurations for measuring this force can be used: one measures the bending force, and the other measures the blocked force, which is higher than the bending force. Studies where the bending force is evaluated report mean values of 4.5 N at 407 kPa in silicone actuators with a length of 80 mm. Silicone actuators of different lengths are also compared, and shorter actuators are shown to generate more force, e.g., a 60 mm long silicone actuator achieves the maximum force (5.58 N) at 450 kPa (Sun et al., 2017). Research on hydraulically actuated FR silicone actuators shows generated forces of 9 N at the tip of the actuator. This study likewise varies the actuator stiffness by temperature, which modifies the force performance of the actuator (Peters et al., 2019). In 2016, silicone actuators for rehabilitation or assistive hand devices were compared. This study obtained bending force values for actuators of different elastomer references, such as actuators constructed with Dragon Skin 10 achieved values of 3.19 N at 180 kPa and actuators constructed with Dragon Skin 20 achieved 3.5 N 380 kPa (Yap et al., 2016). Of the few studies that characterize textile actuators, an experiment was found comparing textile actuators with and without pleats based on the torque generated, which showed that the actuators with pleats built-in that study achieved values of 207.8 N.mm at 172.4 kPa, while the actuators with pleats generated 171.4 N.mm at the same pressure Cappello et al. (2018). According to this study, it is concluded that the difference in the results is contributed to the pleats in the outer layer, as they are the only difference between these actuators.

The blocked force test is used more frequently to characterize these types of actuators, so more information is available for comparison. The force recorded in this test is usually higher than the force recorded in the bending force test. For example, in a 13 cm long PneuNets silicone actuator, a blocked force of 1.2 N was generated at only 43 kPa (Polygerinos et al., 2013). Blocked force values of 1 N at 200 kPa were reported in FR actuators for Dragon Skin 10 silicone, and for references such as Elastosil M4601, values of 5 N were obtained at pressures of 400 kPa. These actuators were built to measure 17 cm in length (Galloway et al., 2013). Likewise, for these same types of FR actuators, values of 8.8 N at 180 kPa were achieved for materials such as Dragon Skin 10 and 9.96 N at 380 kPa for Dragon Skin 20 (Yap et al., 2016). Values of 9.12 N at only 120 kPa were achieved in a silicone and textile hybrid actuators (Yap HK. et al., 2017).

These tests are an easy way to compare actuator designs and generate relevant information for the development of wearable devices. Although standard for characterizing silicone actuators, they have not been applied to textile actuators. Therefore, this study seeks to compare a FR-type silicone actuator and a textile actuator with pleats to determine which type of actuator is mechanically better for the creation of a hand exoskeleton. The selection of the pleated actuators over the classical textile actuators was made by preliminary literature review Cappello et al. (2018) and own laboratory experiments. The key selection criteria was that the pleated actuators achieve the total bending angle faster and require less pressure, which makes them more viable in the construction of a hand exoskeleton (Polygerinos et al., 2013).

## 2 Methodology and Materials

**FIGURE 1:**
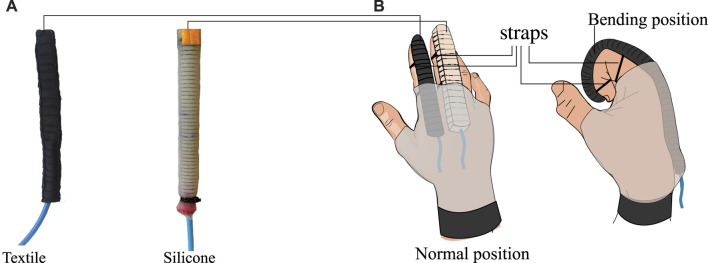
**| (A)** Pleated textile actuator (left side) and Fiber-reinforced silicone actuator (right side) constructed for comparison in this study. **(B)** Possible placement of soft bending actuators in an assistive or rehabilitation glove.

Three tests were performed to compare five textile pleated actuators and five FR silicone actuators (see Figure 1A). The selected tests characterize the actuators according to the relevant properties for designing a device with soft actuators. Table 1 presents the relevant requirements against which the test results will be evaluated. Each parameter in the table was defined based on engineering criteria and clinical contributions.

**TABLE 1:** | Design requirements for the construction of a soft glove for hand rehabilitation or assistive.

Characteristic	Requirements
Weight of Glove	<0.5 kg
Weight of Actuator	<100 g
Total Weight	<3 kg
Width	<2 cm
Minimum bending angle	250°
Speed of actuation	1–3 s
Force for assistive	∼ 7 N
Force for rehabilitation	∼ 3 N

In this study, five textile actuators with pleats and five FR-type silicone actuators were constructed to generate the bending motion necessary to assist in the closing movements of the fingers in an hand exoskeleton. Figure 1B shows how actuators with this type of motion can be positioned for use in a hand exoskeleton. These actuators were designed to have a length of 16 cm and a width of 2 cm. The other dimensions depend on the manufacturing method and materials of construction.

### 2.1 Fiber-Reinforced Silicone Actuator

This type of actuator is built by pouring elastomeric materials such as silicones into 3D printed molds. Depending on the type of motion generated, reinforcements are made with rigid carbon fiber layers and inelastic thread (Polygerinos et al., 2015b). The manufacturing process consists of five phases: 1) pouring the elastic silicone into a mold with a half-circumference cross-section, 2) placement of reinforcements for motion generation, 3) coating the reinforcements with a silicone and 3D mold, and 4) sealing of tips and 5) adding the tubing for inflation. The materials used for constructing this type of actuator were: Dragon Skin 30 Part A and B, an internal mold, an external mold, inelastic thread, carbon fiber, and an end sealing mold. The relevant mechanical properties of the material can be seen in Table 2.

**TABLE 2:** Material properties of silicone actuator construction.

Material	Tensile strength	Modulus	Elongation at break
Dragon Skin 30	500 (psi)	86 (psi)	364 (%)

**FIGURE 2:**
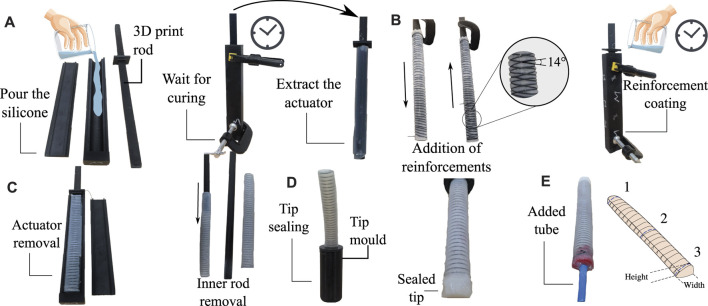
The construction process of an FR type silicone actuator. **(A)** First stage: pouring the silicone into the internal mould, pressing the mould to avoid leakage during curing and removing the actuator after silicone curing. **(B)** Second stage: addition of reinforcements for bending movement. Initially, a double spiral is woven, and the inelastic layer is glued to the bottom of the actuator. The reinforcements should be coated with silicone through the external mould. **(C)** Third stage: removal of the actuator from the inner rod. **(D)** Fourth stage: Sealing off the ends. **(E)** Fifth stage: Placement of the plastic tube to inflate the actuator.

The first stage of construction of this actuator was performed by preparing the silicone and pouring it into the internal mold. A half-circumference cross-section bar was inserted to generate the internal chamber of the actuator (see Figure 2A). After the silicone fully cured for 6 hours, the internal mold was separated from the actuator. In the second stage, the reinforcements were added as follows: inelastic thread was woven around the actuator, creating a double spiral starting at the tip and ending at the bottom of the actuator, after which another spiral was made in the opposite direction. This double spiral generates an angle of 14° between each reinforcement thread as shown in Figure 2B. After, the reinforcement of the inelastic carbon fiber layer was glued to the flat part of the actuator with Sil-Poxy (Smooth-On’s, USA). This second stage of construction is illustrated in Figure 2B. The actuator is coated with silicone using the external mold to protect the reinforcements. The next stage consisted of removing the inner rod (see Figure 2C), and finally, sealing the ends of the actuator using the sealing mold (see Figure 2D) and inserting a tube at one of the ends (see Figure 2E). The whole construction process required 2 days because the silicone must cure on 3D molds to achieve the best results. The construction process and measurements of the molds was based on the methods explained on the website Soft Robotics Toolkit Holland et al. (2014). The deformation of this actuator can be seen in Figure 4A, which shows the states of rest and full bending in the fiber-reinforced silicone actuator. One feature of this actuator construction is that different movements can be configured in a single actuator design, such as bending, extension, and torsion. However, this cannot be modified after construction: the actuator will always have the same behavior. An example of this property is the design of a thumb actuator, which integrates different motions in a single actuator (Maeder-York et al., 2014).

### 2.2 Pleated Textile Actuator

The textile actuators uses elastic and inelastic fabric materials and an thermoplastic elastomer (TPE) air-containing element to perform the bending motion. Unlike silicone actuators, this construction process is performed in 2D by stacking layers of fabric (Cappello et al., 2018) and applying pressure on the actuator so that a 3D structure is generated. The construction process is performed in multiple stages. Initially, a layer of stiff fabric is sewn to a layer of elastic fabric to produce a finger-sized pocket. This layer must be stitched, generating folds that facilitate the bending movement (Yap HK. et al., 2017). The materials used for constructing this actuator were: a plastic tube, TPE layer (Stretchlon 200, FibreGlast), an impulse sealer, an industrial sewing machine, rigid fabric, and a Lycra-type elastic fabric (Lycra-Nilon POWER ID-0019-056, Facol, Colombia). The mechanical properties of the selected fabrics are presented in Table 3. These were calculated based on the ASTM D5035 standard test that determines the breaking strength and elongation of a material.

**TABLE 3:** Specifications of the materials used in the development of the textile actuator.

Material	Rupture force (N)	Elongation (mm)
Elastic Fabric	258.47 ± 42.68	411.34 ± 42.67
Rigid Fabric	448.17 ± 35.38	12.220 ± 0.700

**FIGURE 3:**
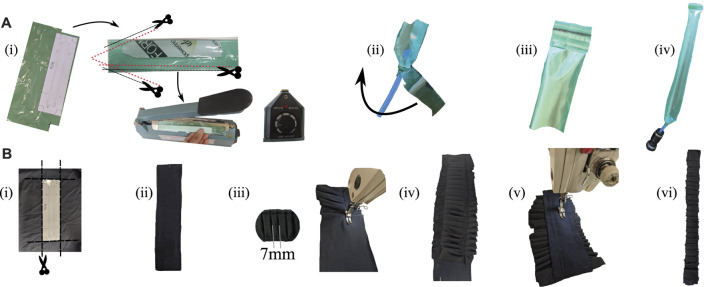
The construction process of a textile actuator with pleats. **(A)** Construction of the internal balloon keeps the air in the actuator; **[(A), i]** Cutting the plastic and sealing with the impulse sealer machine. **[(A), ii]** Placement of the plastic tube for inflation. At this point, plastic reinforcements are added to prevent leakage at the joint. **[(A), iii]** Sealing the end of the actuator several times to avoid leaks. **[(A), iv]** The pressurised balloon is shown. **(B)** Construction of the textile pocket with pleats; **[(B), i,ii]** Cutting the rigid and elastic fabric. **[(B), iii]** Joining the elastic fabric over the rigid one while making the pleats. **[(B), iv]** Pocket with pleats sewn on both sides. **[(B), v]** Definition of the actuator width through a line made with the sewing machine. **[(B), vi]** Cutting out excess fabric from the pocket.

This actuator can be manufactured in two independent processes: 1) the construction of the internal air-containing balloons, and 2) the pleated pockets that generate the bending motion. The TPE inner balloon was constructed following these steps. First, the TPE sheet is cut out and joined with the plastic impulse sealer, as shown in Figures 3Ai. This step ensures the two layers of the sheet are completely fused to avoid air leakage during pressurization. The TPE plastic is then glued to the plastic tube with the help of a specialized glue (Loctite 406, Henkel Adhesives). To ensure a perfect connection without leakage, layers of the TPE plastic were added around the tube, as shown in Figure 3. After the glue solidified, the balloon is inverted to get the hose joint inside the balloon; this also helps avoid air leakage at the time of pressurization. Finally, the missing end is sealed with the impulse sealer, as shown in Figure 3Aiii.

To carry out the construction of the outer pocket of the textile actuator, fabrics were initially cut out as shown in Figure 3Bi and Figure 3Bii then sewn together with the sewing machine. The elastic layer must be joined to the rigid layer at this stage while the pleats are made in the elastic fabric (Figure 3Biii). The pleats of these actuators were made 7 mm from tip to tip as shown in Figure 3Biii. However, the construction process, being more dependent on human skill, does not preserve the uniformity of this measurement in each pleat. This is one of the reasons why five actuators of each type were built to average the performance of these actuators. Once this is done (Figure 3Biv), a line defining the actuator width is sewn, as shown in Figure 3Bv. Finally, the excess fabric is trimmed off (Figure 3Bvi).

**FIGURE 4:**
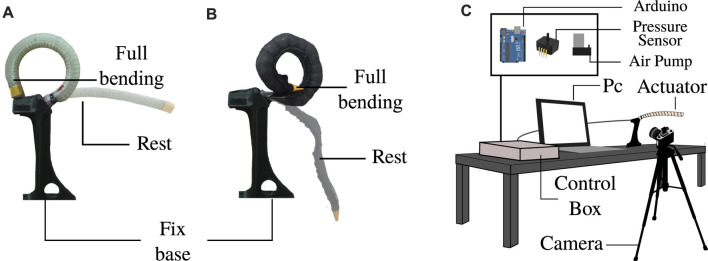
Test measurement of the pressure required to achieve full bending. **(A)** Definition of full bending in the textile actuator and **(B)** the silicone actuator. **(C)** Setup of the key elements to perform the test measurement.

The construction time of a textile actuator with pleats can take approximately 2 h. The deformation of this actuator can be seen in Figure 4B, which shows the states of rest and full bending in the pleated textile actuator. The advantage of this actuator is that it can generate different movements with the addition of another layer and another internal balloon. However, unlike silicone actuators, these actuators can generate independently controlled bending and extension movements (Yap HK. et al., 2017), (Cappello et al., 2018).

Although the properties in Tables 2 and 3 are not directly comparable, they are related within their respective class. For example, these properties are among the stiffest in silicone soft actuator construction, with Dragon Skin 30 being stiffer than other elastomers such as Ecoflex 00-30 or Ecoflex 00-50 (Marechal et al., 2020). Likewise, the stiffest of three elastic fabrics was used. These materials were selected from an earlier preliminary study based on their increased durability in actuators for wearable device applications.

### 2.3 Physical Test

The first test involves measuring the weight, width, and height of the actuators. Each actuator type was built with the same length of 16 cm, and the width measurement was designed to be 2 cm for a fair comparison. However, because of the different construction methods and experience with the manufacturing process, this measurement is variable for the study. Height is an essential variable of the actuators to compare because it can establish which type of actuator takes up less space and is more comfortable for the wearer. Finally, the weight of the actuators was measured to determine which was the lightest.

The weight of the actuators was measured with a digital balance having a resolution of 1 g. Five measurements were taken for each actuator and averaged. For dimensions, width and height were measured three times per actuator. The values were taken in three sections along the actuator to analyze the construction and manufacturing processes (Figure 2E). These measurements were taken in two states, depressurized and pressurized. This state variation shows how the dimensions of the actuators change when they are operated.

### 2.4 Pressure for Full Bending Angle

The second test to compare the actuators was based on identifying the air pressure required to achieve a full bending motion. This test provides information on the efficiency of the actuator: the less pressure required, the more efficient the actuator is considered to be in a portable pneumatic device. This premise suggests that the actuator requires less pressure to be actuated and thus uses smaller air devices, reducing the weight and size of the overall system. The test performs five repetitions in five actuators of each type (textile-silicone), where the actuators change from the depressurized state to the complete bending position. The full bending angle is considered successful when the tip of the actuator touches the top of the fixed base in the configuration, as seen in Figures 4A,B. At this point of full bending, the pressure value captured by the pressure sensor was acquired by the simultaneous recording of the actuator and the pressure recorded on a monitor screen (Figure 4C). The air pressure measurements were performed using the ASDXACACX100PAAA5 sensor (Honeywell, USA) and an Arduino UNO for acquisition. All test angle behavior was recorded with a 60 fps camera. Computer vision processing was performed with the open-source software Kinovea.

### 2.5 Full Bending Time

Another parameter compared in this study was the time it takes for the actuator to move from the resting state to full bending. Using the same setup as the previous test (Figure 4C), the time required to reach full bending was acquired by processing the video with Kinovea software. Five repetitions were performed for each actuator; five silicone actuators and five textile actuators. Each of the repetitions consisted of pressurizing the actuator at maximum pump flow and recording the behavior through the camera. Air was injected with a 32 psi electric pump at 40 L per minute airflow rate using an air pump (ZH712-8504-5000, China). This test shows which actuator reaches the full bending position the fastest.

### 2.6 Bending Force and Block Force

**FIGURE 5:**
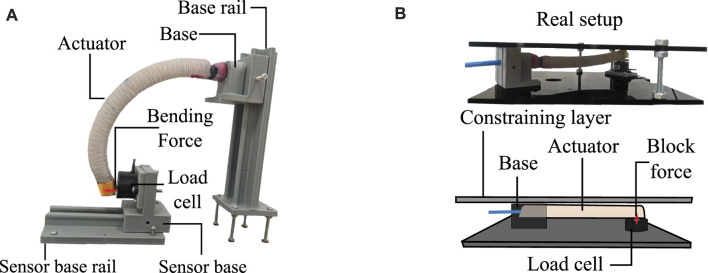
Test of force exerted by the actuators. **(A)** Configuration for bending force measurement. It can be seen how the clamping system of the sensor and the actuator can modify its location through rails to adjust the tip of the actuator with the load cell. **(B)** Configuration for blocked force measurement. The upper image shows an actual image of the test. The lower image shows a graphical explanation showing the key elements of the setup.

Finally, the last comparison test performed measured the forces the actuator can generate through the full bending test. For this experiment, two different setups were used. These tests are shown in Figure 5, with part (a) demonstrating the determination of the bending force and part (b) finding the blocked force of the actuators.

The sensor used to perform the force measurement was an FC2211-0000-0010-L load cell (TE Connectivity, Switzer-land). The sensor data acquisition was performed with an Arduino UNO. This test was performed five times per the ten actuators as in previous tests.

### 2.7 Data Analysis

The statistical analysis of the data was performed in two ways: 1) descriptive statistics to organize and visualize the data graphically based on the mean, deviation, and coefficient of variation of the results, and 2) inferential statistics to find the relevant differences between the two actuators in each test performed. For these inferential analyses, the normality of the data was verified using the Shapiro-Wilk test. The two variances between the classes were analyzed according to the F-test, depending on the normality of the test results. The purpose of this analysis is to compare the means of the two categories and determine if there are significant differences between the two actuators. The inferential tests used were the Mann-Whitney test and T-test, according to the normality of the data. The results of these inferential tests are represented by the *p*-value (p), with a confidence level of 5%. This means that if the *p*-value is less than 0.05, the results in comparison are considered to have significant differences. The statistical analysis was implemented in Excel and RStudio software.

## 3 Results

### 3.1 Physical Test

The textile actuators weighed 7.12 ± 0.92 g, and the silicone actuators weighed 32.52 ± 1.44 g. The actuators were built to have the same length and width, so the weight variation is a result of the construction materials, with the silicone having a higher density and volume compared to the lighter textile layers.

**FIGURE 6:**
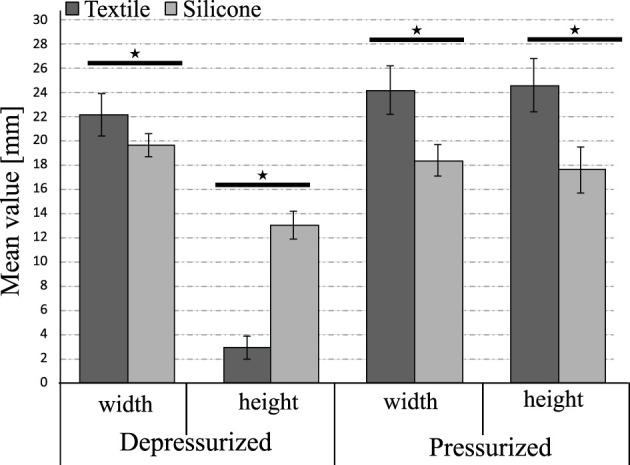
Comparison of critical dimensions for wearable devices and their variation according to the actuation state in silicone and textile actuators. The symbol ⋆ represents a significant difference between the two groups based on statistical analysis tests (*p* < 0.05). For all variables, there are significant differences in the change of state (depressurised, pressurised).

Figure 6 shows the results of the physical variables measured in the pressurized and depressurized states. This test illustrates how the dimensions vary between the two states and how the manufacturing method affects the results. For example, the actuators’ width was designed to be 20 mm. However, both the textile and silicone actuators do not have that exact value as shown in the results. In the depressurized state, the construction method that comes closest to having the designed value is the 3D printing method for silicone actuators (19.644 ± 0.98 mm), as opposed to the result obtained for the textile actuator (22.17 ± 1.73 mm). Although the resulting values of the two types of actuators are close, there are significant statistical differences between them, indicating the silicone actuator construction method is more accurate for the variable “width”.

Due to the 2D construction of the textile actuators, which is based on piling up layers with different properties, the textile actuators have a height of 2.92 ± 0.93 mm when placed on the exoskeleton, as shown in Figure 1B. In contrast, the unactuated silicone actuators measures 13.012 ± 1.13 mm. In the pressurized state, the textile actuators is larger than the silicone actuator, both with regards to width and height. Specifically, the textile actuators would measure 24.16 ± 1.99 mm above the human finger when the exoskeleton is in its actuated state, compared to 18.35 ± 1.3 mm for the pressurized silicon actuators. This behavior is caused by the materials of construction rather than the manufacturing method. When using material such as Lycra that deforms in several directions, the elastic layer expands in two directions when the textiles actuators are pressurized. One layer is responsible for generating the bending movement, and the other generates an increase in the actuator width dimension. Meanwhile, the silicone actuators have reinforcing elements, such as inelastic thread, that permit the redirection of the actuator’s deformation. This allows reorienting the deformation of the material in only one direction, which enables the actuator to bend without increasing the width of the actuator.

**FIGURE 7:**
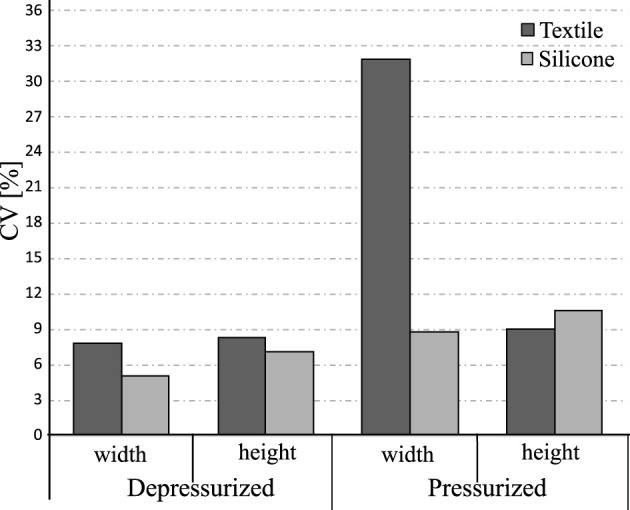
The coefficient of variation calculated based on the three sections of the actuator (top, middle and bottom). The higher CV value means less accurate is the manufacturing method of the actuator.

To evaluate the accuracy of the manufacturing methods for each actuator type, the coefficient of variation (CV) was calculated for the three sections measured on the actuator. This statistical calculation provides information about how constant and uniform the measurements are throughout the whole of the actuator. Figure 7 shows that although the CV calculated with the three measured sections is low for the two construction methods, it is higher for the textile actuators compared to the silicone actuators. Likewise, the CV generated by the 3D printing manufacturing method based on molds is more accurate than using industrial machines to sew the textile actuators (5.019% for the silicone actuator and 7.83% for the textile actuator).

The result suggests that the silicone actuators in its depressurized state has a uniform height throughout the actuator. In contrast, the textile actuators has pleats in the top layer, generating variation in height throughout the actuator.

When the actuators are pressurized, the CV of the width of the silicone actuators is greater than its depressurized state. The textile actuators show significant variations in the measured sections when pressurized. These variations can be associated with the manufacturing method and the sewing skill of the builder. To avoid variations in the dimensions, it is necessary to have adequate precision and sewing machine experience to compete with the technology and accuracy of a 3D printer. Finally, the CV of the height of the textile actuators in the pressurized state is low than the silicone actuators.

### 3.2 Pressure for Full Bending Angle

**FIGURE 8:**
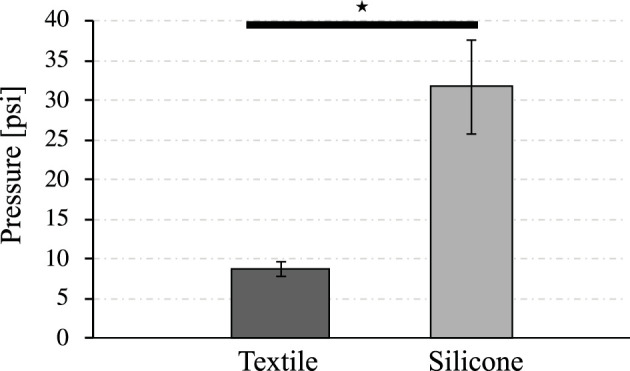
Air pressure needed to achieve full bending performance for both types of actuators. The symbol ⋆ represents a significant difference between the two groups based on statistical analysis tests (*p* < 0.05).

Figure 8 shows the pressure required for the actuators to achieve the full bending motion based on the data acquired in the 25 samples for each type of actuators. The textile actuators achieve the maximum bending angle at a value of 8.74 ± 0.93 psi (60 kPa), and the silicone actuators at a higher value of 31.6 ± 5.8 psi (213 kPa). This indicates that both types of actuators can be used with small air sources and low power. The difference between the actuator types is that the silicone actuator requires 261% more pressure compared to the textile actuator. Therefore, thanks to the inferential analysis, there is a statistically significant difference between the two types of actuators. It can be concluded that the textile actuators are more energy efficient to achieve the maximum bending angle with less pressure. Although the silicone actuator meets the test objective and the requirements set out in the Table 1, it is less efficient than the textile actuators.

### 3.3 Full Bending Time

**FIGURE 9:**
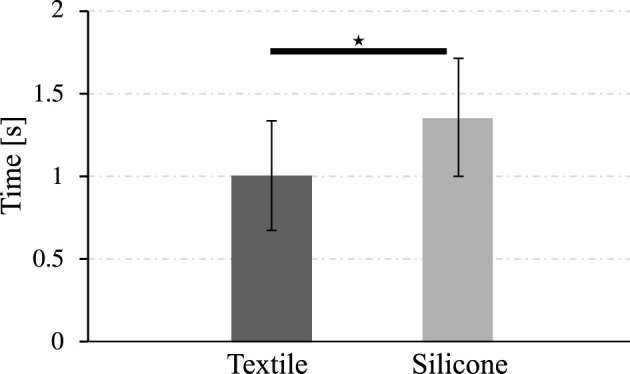
Time required by the actuators to reach full bending position. The symbol ⋆ represents a significant difference between the two groups based on statistical analysis tests (*p* < 0.05).

Figure 9 shows the time required by the actuators to achieve full bending. The textile actuators with pleats achieves full bending in 1.01 ± 0.33 s, while the silicone actuator takes 1.35 ± 0.35 s. The standard deviation in the data is similar between the two actuators, so the repeatability of the time required to reach full bending is similar for both, indicating that the textile actuator is consistently quicker to reach this state. According to the inferential analysis, it is confirmed that there are significant differences between the time it takes for the actuators to reach full bending. Thus confirming that the textile-type actuators are faster than the silicon actuators.

### 3.4 Bending Force and Block Force

**FIGURE 10:**
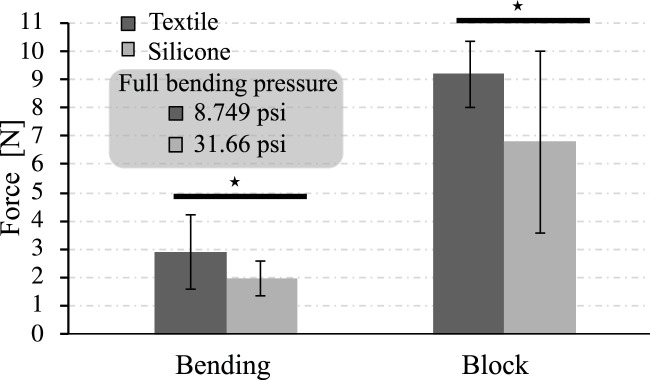
The force generated by the actuators according to the two different setups and the comparison full bending pressure. The symbol ⋆ represents a significant difference between the two groups based on statistical analysis tests (*p* < 0.05).

Similar results were obtained from the bending force test with regards to the force exerted. The textile actuators generated an average peak force of 2.90 ± 1.31 N at 8.749 psi and the silicone actuators 1.96 ± 0.62 N at 31.6 psi (see Figure 10). If the force exerted at the actuator’s tip is compared at the same input pressure, the silicone actuator is shown to have a less force. This indicates the textile actuators are more efficient than the silicone actuators.

Similarly, the blocked force test results show that the textile actuators generate more force than the silicone actuators. In this case, the textile actuators generate 9.18 ± 1.16 N of blocked force, far higher than the silicone actuators’ 6.78 ± 3.2 N.

There is a statistical difference between the force generated between the two types. The textile actuators generate greater forces than the silicone actuators in the blocked force configuration. Another critical point seen in Figure 10 is the deviation of the data in the silicone FR-type actuator. The data are more dispersed in this actuator type in the blocked force test. This affirms that the behavior of the silicone actuator in terms of forces at the actuator tip is less consistent and more complex to control than that of the textile actuators.

## 4 Discussion

The results were analyzed to determine which type of actuator performed better with regards to weight, actuator dimensions, manufacturing processes, efficiency, time, and force to construct and adapt a biomedical device according to the design requirements. Both types of actuators meet the weight requirement defined in Table 1. However, the actuators constructed with the textile technique are 78% lighter than the silicone actuators. Assuming that the device’s weight is only dependent on the weight of the actuators, it can be estimated that an exoskeleton built using textile actuators would weigh approximately 15 g. Compared to the tendon-based exoskeleton’s 69 g, the textile device’s weight is significantly lower (Tran et al., 2020). The results of this test suggest using textile actuators if the device’s weight is a factor in the construction of an exoskeleton.

In terms of actuator dimensions, the results of Figure 6 suggest using silicone actuators if the application requires only minor modifications in dimensions due to pressurization. Similarly, the results in Figure 7 suggest using these actuators if precision in the construction method is essential. Compared to textile actuators, silicone actuators have more constant dimensions throughout due to their manufacturing method based on 3D printed molds. In contrast, it is more feasible for a hand exoskeleton to use textile actuators with pleats since they take up very little space in their resting state and weigh very little.

Figure 6 illustrates which type of actuator deforms more when pressurized. If the actuator type is selected based on which one occupies less space without sacrificing functionality, the width of the pressurized silicone decreases compared to its depressurized state, which is an advantage in this type of application by reducing the space used by the actuator within the system. This is in contrast to the textile actuators, which increase their width by about 8.9% when pressurized. In both cases, the differences between the dimensions are statistically significant. Finally, the height variation between the depressurized and pressurized state of the textile actuator is 741% higher than its initial value, which is greater than the 35% increase in the silicone actuator.

Although the results of Figure 7 show that the fabrication method of the textile actuators is less accurate, this variation is not as relevant in this application. No dimension generates values greater than 35% in the calculated coefficient of variation based on the measured sections. This indicates that the fabrication method of the textile actuators does not generate significant variations in the final dimensions. The results of Figures 6, 7 indicate that either actuator compared in this study can be used for the construction of a hand rehabilitation and assistance device with regards to dimensions and manufacturing precision.

Since both types of actuators can be used in constructing a hand exoskeleton based on dimensions and weight, it is essential to evaluate which one has better performance regarding the energy required to achieve the full bending motion. Figure 8 shows textile actuators require 72.3% less pressure than silicone actuators to perform the full bending motion, allowing this type of actuator to be used and operated with low power and smaller systems. This reduction in the power system for rehabilitation and assistive devices is essential to reduce costs and the overall associated weight, which contributed to the development of wearable and portable systems.

Compared with the results presented above, FR-silicone actuators achieve the full bending motion around 213–250 kPa (Galloway et al., 2013), (Polygerinos et al., 2015b), (Ariyanto et al., 2018). These values confirm that the behavior of the constructed actuator coincides with the values determined in other studies. For actuators built with Elastosil M4601, a 16 cm actuator achieves the maximum bending at 243 kPa (Galloway et al., 2013). However, this material has a value of 945 psi of tensile strength, which is higher than the Dragon Skin 30. A 16 cm FR actuator achieved maximum bending at 200 kPa (Polygerinos et al., 2015b), as did the silicone actuator in this study. Although not directly comparable, the actuator achieved half bending at 110 kPa in a study where silicone actuators were built for the construction of a hand exoskeleton, confirming that the maximum bending of these actuators is within the range of 200–250 kPa (Ariyanto et al., 2018). Based on existing literature and the results presented in the current study, textile actuators require less air than FR-type silicone actuators to achieve full bending. This property makes the textile actuators a better choice in constructing a hand exoskeleton if pneumatic efficiency is a factor.

In addition to being more energy-efficient, textile actuators require 25% less time than fiber-reinforced silicone actuators to reach full bending, as shown in Figure 9. According to the requirements in Table 1, both types of actuators are within the defined actuation speed range. Although both actuators meet the defined time of less than 20 s from the literature (Li et al., 2019), (Borboni et al., 2016), the textile actuators are better in terms of actuation time and meeting the requirements.

The results of Figures 8, 10 illustrate that textile actuators generate more force than silicone actuators at lower pressures, indicating their use will be more beneficial for the construction of a rehabilitation or assistive device. As with the pressure required to achieve maximum deflection, the FR silicone actuators constructed in this study returned values similar to those reported in other studies, e.g., block force values of 1 N at 200 kPa were reported for an FR-type silicone actuator built with Dragon Skin 10 (Galloway et al., 2013). The results of the bending force in this study similar compared to the results presented by other studies. Values of 3 N at only 180 kPa were achieved in this test with a silicone actuator built with Dragon Skin 10 and 3.5 N at 380 kPa with one built with Dragon Skin 20 (Yap et al., 2016). These differences in pressures and slight variation in force indicate that the material affects the force generated in silicone actuators. The more rigid the material, the more pressure will be necessary and the less force it can exert. As a result, this study’s actuators built with Dragon Skin 30 achieve 2 N at the bending pressure (213 kPa). From the results obtained and compared with state-of-the-art silicone actuators, it is inferred that more air pressure should be applied to increase the force exerted by the FR-type silicone actuators, or the construction material should be changed to provide less resistance and more elasticity.

According to the requirements defined in Table 1, and the results demonstrated in Figure 10, the silicone actuators used in this study does not achieve the minimum force required to be functional in rehabilitation or assistive device (Agarwal et al., 2016). The textile actuators exceeded the 3 N defined in the design requirements. It is important to note that the force obtained in this study is the force generated at the pressure where maximum bending is performed. The maximum force that the actuators can achieve would be found by pushing the actuators until they fail mechanically, i.e., the device has a rupture and air escapes, generating pressure losses. It is also important to note that the results obtained in this study are the resulting discrete values at the specific bending point since this is the one that interests us in the construction of a hand exoskeleton. However, this comparative study can be complemented by analyzing the behavior of angles vs. different pressure levels and seeing the resulting forces at different input pressure values.

Overall, the actuators constructed with the pleated textile technique have greater potential in the construction of a portable device for hand rehabilitation and assistance functions. Although they occupy more space than silicone actuators when pressurized, characteristics such as weight, the force generated, and efficiency are much better than silicone actuators. The dimensions can also be modified into a smaller actuator if necessary, which is likely to further reduce the weight and require less energy to operate. In addition, this actuator allows for an extension motion by adding a rigid layer of fabric and an internal balloon. These modifications do not add much weight to the device, and they enhance the chances of rehabilitation. In comparison, adding the extension movement to the silicone actuator would necessarily double its weight and dimensions.

Although not part of the initial design requirements, it is worth noting that textile actuators are easier to repair compared to silicone actuators. To repair a textile actuator, it is simply necessary to replace the internal balloon with one in good condition. In silicone actuators, if the actuator cannot be repaired with products such as Sil-PoxyTM or Smooth-On, it must be built from scratch to correct the fault in the device. This would involve more time and material than the textile actuator. The lifecycle of soft actuators will be analyzed in future work because it is vital to improve the number of cycles that the actuator can generate during a rehabilitation session without replacing any parts or the actuator itself.

## 5 Conclusion and Future Work

This study focused on comparing the physical properties, the efficiency, air pressure requirements, and the force generated by two types of soft actuators. These variables are essential in deciding which type of actuator to use in the construction of a hand exoskeleton. The fabrication methods for silicone actuators were identified as more time consuming but more dimensionally accurate than textile actuators. Although the silicone actuators do not have considerable changes in dimensions due to state changes, it is recommended that textile actuators are used to build the device because they are 78% lighter, require 72% less pressure to be actuated, and generate 48% more bending force and 35% more block force than the silicone actuators. These improvements in the performance of pleated textile actuators are contributed to the fact that the material of construction has lower density and is, therefore, lighter, properties such as material elongation in textile materials are higher, and thus, Young’s modulus allows these actuators to deform with less pressure than silicone actuators. Finally, thanks to the pleats and how these actuators generate the full bending, the geometry allows obtaining more force than fiber-reinforced silicone actuators.

Although both types of actuators satisfy the basic requirements for the use and construction of a rehabilitation or assistive hand exoskeleton, the textile actuator can reduce the system’s overall size because the air pump can be of a smaller size and require less power. As evidence, it is assured that the advantages presented for pleated textile actuators over silicone fiber-reinforced actuators are for these specific models and materials. However, it can be assumed that the benefits presented can be general for any type of textile actuator since these will always be lighter and require less pressure to operate. In addition, these actuators allow for easy repair and maintenance, ultimately reducing the manufacturing cost because the entire actuator does not need to be replaced for repair. The materials are likewise low in cost and easy to procure.

In the future, experiments in which force and angle profiles can be identified at different pressures will be performed, and the lifecycle of the actuators will be studied to determine improvements that allow rehabilitation therapies to proceed without requiring replacement or repair of the actuators. In addition, an assistive hand device will be built with the textile actuator as a result of the efficiency displayed in the tests discussed here. Functional tests will be performed on healthy patients using this device to evaluate its performance.

## Data Availability

The original contributions presented in the study are included in the article/Supplementary Material, further inquiries can be directed to the corresponding author.
